# Integration of SuperCam based chemical imaging and clustering to correlate geochemistry and mineralogy in heterogeneous samples

**DOI:** 10.1038/s41598-025-21770-4

**Published:** 2025-10-29

**Authors:** Laura García-Gómez, Iratxe Población, Tomás Delgado, Javier Laserna, Marizú Velásquez, Pavel Porizka, Jakub Buday, Jozef Kaiser, Álvaro Lobato, Ana I. Casado, Mercedes Taravillo, Juan Manuel Madariaga, Julene Aramendia, Fernando Rull, Daniel Carrizo, Jesús Martínez-Frías, Iñaki Vadillo, José Miguel Vadillo, Francisco J. Fortes, Luisa M. Cabalín

**Affiliations:** 1 UMALASERLAB, Departamento de Química Analítica, Universidad de Málaga, Málaga, Spain; 2 Department of Analytical Chemistry, University of the Basque Country (UPV/EHU), Leioa, Spain; 3 Instituto Universitario de Materiales y Nanotecnología, IMANA, Universidad de Málaga, Málaga, Spain; 4 Central European Institute of Technology, Brno University of Technology, Brno, Czech Republic; 5 Faculty of Mechanical Engineering, Brno University of Technology, Brno, Czech Republic; 6 Department of Physical Chemistry, Universidad Complutense de Madrid (UCM), Madrid, Spain; 7 ERICA Research Group, University of Valladolid (UVa), Valladolid, Spain; 8 Centro de Astrobiología (CSIC-INTA), Madrid, Spain; 9 Instituto de Geociencias (IGEO, CSIC-UCM), Madrid, Spain; 10 Grupo de Hidrogeología, Departamento de Ecología y Geología, Facultad de Ciencias, Universidad de Málaga, Málaga, Spain

**Keywords:** LIBS, Mapping, K-means, Planetary exploration, LIBS, Optical techniques, Planetary science

## Abstract

Peridotites are ultramafic rocks whose mineralogical diversity makes them suitable case studies for testing analytical methodologies. In this study, a peridotite sample from the Ronda massif (Málaga, Spain) was examined demonstrating a practical methodology for distinguishing geochemically distinct regions within complex lithologies. The sample was analyzed using a multi-instrumental approach combining laser-induced breakdown spectroscopy (LIBS), micro-energy dispersive X-ray fluorescence (µ-EDXRF), and Raman spectroscopy, all of them integrated in the Perseverance rover payload. LIBS and µ-EDXRF were used to assess elemental composition and spatial distribution across the sample, whereas Raman spectroscopy confirmed the presence of mineral phases such as, olivine, pyroxenes, and chromiferous spinels in specific regions. Elemental LIBS ratios such as Mg# and Cr# further supported the identification of compositional variations related to mineral phases. Spectral LIBS data were processed using k-means clustering to segment geochemical zones and detect spatial trends. This integrated spectroscopic and statistical approach enables interpretation of ultramafic rock complexity and offers an efficient framework for planetary exploration under mission constraints.

**Supplementary Information:**

The online version contains supplementary material available at 10.1038/s41598-025-21770-4.

## Introduction

Peridotites are ultramafic igneous mantle-derived rocks dominated by olivine and pyroxene. Although peridotitic outcrops have not been identified on Mars, their main constituent minerals have been detected on the planet’s surface through a combination of remote sensing and in situ techniques. Remote sensing instruments including OMEGA (Observatoire pour la Minéralogie, l’Eau, la Glace et l’Activité), and CRISM (Compact Reconnaissance Imaging Spectrometer for Mars) imaging spectrometers, have identified diagnostic spectral features in the near-infrared range, in regions such as Nili Fossae, Valles Marineris, Syrtis Major, and Jezero crater^1,2^. In parallel, the Perseverance rover has also confirmed these findings, with its combined approach with different instruments, SuperCam has allowed to combine elemental and molecular information using laser induced breakdown spectroscopy (LIBS), Raman and VISIR/IR reflectance spectroscopy, while the Remote Micro-Imager (RMI) has served to spatially contextualize the data obtained from the Martian surface. Furthermore, SHERLOC (Scanning Habitable Environments with Raman & Luminescence for Organics & Chemicals) has assisted mineralogical information by Raman and fluorescence data, mainly for the detection of hydrated and possible organic-bearing. Likewise, PIXL (Planetary Instrument for X-Ray Lithochemistry), through micro-X-ray fluorescence (µ-EDXRF), is able to mapelemental chemistry at sub-millimeter scale^3,4^. This multi-instrument strategy has also refined mineral identification, thereby revealing the existence of secondary alteration phases in certain olivine outcrops, including carbonates and serpentines, minerals also present in altered peridotites^5–7^.

In addition to the main phases mentioned above, peridotites frequently exhibit accessory minerals such as spinels, including Cr-bearing varieties like chromite. These mineral species are of particular interest because they have been identified in Martian meteorites, especially within the shergottite group, and may provide insights into primitive igneous domains^8–10^. However, their direct detection on the Martian surface remains challenging due to spectral limitations and the spatial resolution of current remote sensing techniques. Nevertheless, analyses of LIBS performed by the SuperCam instrument have revealed localized chromium enrichments that may be associated with these mineral phases^3^. Several studies have also underscored their capacity to preserve paleomagnetic signals and to serve as tracers of high-temperature magmatic processes^11^.

The Ronda massif (southern Spain) hosts one of the largest exposed peridotite bodies on Earth and has been extensively studied. Beyond their primary mineralogy, these peridotites exhibit aqueous alteration processes that generate a diverse mineral assemblage and can lead to the preservation of indigenous carbon matter—phenomena also identified in Martian meteorites such as Nakhla^12^. This natural heterogeneity makes the Ronda peridotite a particularly suitable case for assessing the applicability of combined analytical techniques in complex lithologies.

Furthermore, multiple studies have employed terrestrial peridotites to model partial melting, magmatic differentiation, and hydrothermal alteration processes on Mars^13,14^ as well as to validate in situ characterization tools using samples representative of Mars-relevant geological contexts^15–17^.

In case of laboratory terrestrial studies, geochemical characterization of peridotites involves the use of advanced analytical techniques. Commonly, systems such as scanning electron microscopy (SEM) and transmission electron microscopy (TEM) are employed, enabling a detailed examination of the morphology and mineralogy of the sample^18,19^. Other analytical techniques that are well suited for these studies include inductively coupled plasma mass spectrometry (ICP-MS)^20,21^, X-ray diffraction (XRD)^22^ and electron backscatter diffraction (EBSD)^23^. However, due to the extreme conditions of Mars exploration, the available techniques for studying the Martian surface are more limited.

Planetary missions have relied on miniaturized spectroscopic systems capable of performing non-destructive, in situ analyses. Techniques such as LIBS and Raman spectroscopy, implemented in SuperCam and SHERLOC respectively, provide the ability to detect elemental composition and identify mineral phases under Martian environmental conditions. While these instruments offer substantial analytical capabilities, the geological complexity and heterogeneity of planetary samples present significant challenges for direct spectral interpretation. In this context, the application of chemometric tools and classification algorithms has emerged as a powerful strategy to extract meaningful geological information from large and complex spectral datasets. These algorithms can be divided into supervised and unsupervised approaches ^24^. While supervised methods like support vector machines (SVMs) rely on labeled data to classify new samples, unsupervised methods such as k-means clustering do not need prior labels, making them especially valuable for analyzing datasets with limited or no information^24,25^. This is particularly useful for LIBS data, which is highly sensitive to matrix effects and typically requires a large number of standards for supervised methods to be effective^26–28^.

In this work, LIBS was used to generate two-dimensional distribution maps of both major (e.g., Mg, Fe, Si) and minor elements (e.g., Cr, Ni, Al, Na, Sr), as well as to detect carbon through both atomic and molecular emission features. These maps allowed the identification of textural features such as veins and localized alteration zones. In parallel, intensity-based geochemical ratios, including Mg# (Mg/(Mg + Fe)) and Cr# (Cr/(Cr + Al)), were used to support the interpretation of mineralogical domains and to infer the potential presence of phases such as olivine, pyroxenes, and chromium-enriched spinels.

Furthermore, the elemental distributions obtained from LIBS were validated with complementary µ-EDXRF and Raman spectroscopy, confirming the compositional variability and mineral assemblages. Combined with k-means clustering, these datasets allowed the discrimination of chemically distinct regions, improving the identification of potential mineralogical units. Building on this, a methodological approach was developed to characterize the mineralogical and geochemical variability of a peridotite sample from the Serranía de Ronda, as a test case for strategies applicable to planetary exploration. By combining these datasets with automated classification methods, an approach is demonstrated that is particularly well suited to long-duration missions such as Mars 2020 or Mars Science Laboratory, where large volumes of spectral data are acquired. This framework enables preliminary geochemical subdivision without requiring prior mineralogical knowledge, supports target selection for further analyses, and facilitates the comparison of measurements collected across different sols, thereby addressing key challenges of remote planetary data acquisition.

## Sample

### Ronda peridotite sample

**Fig. 1:**
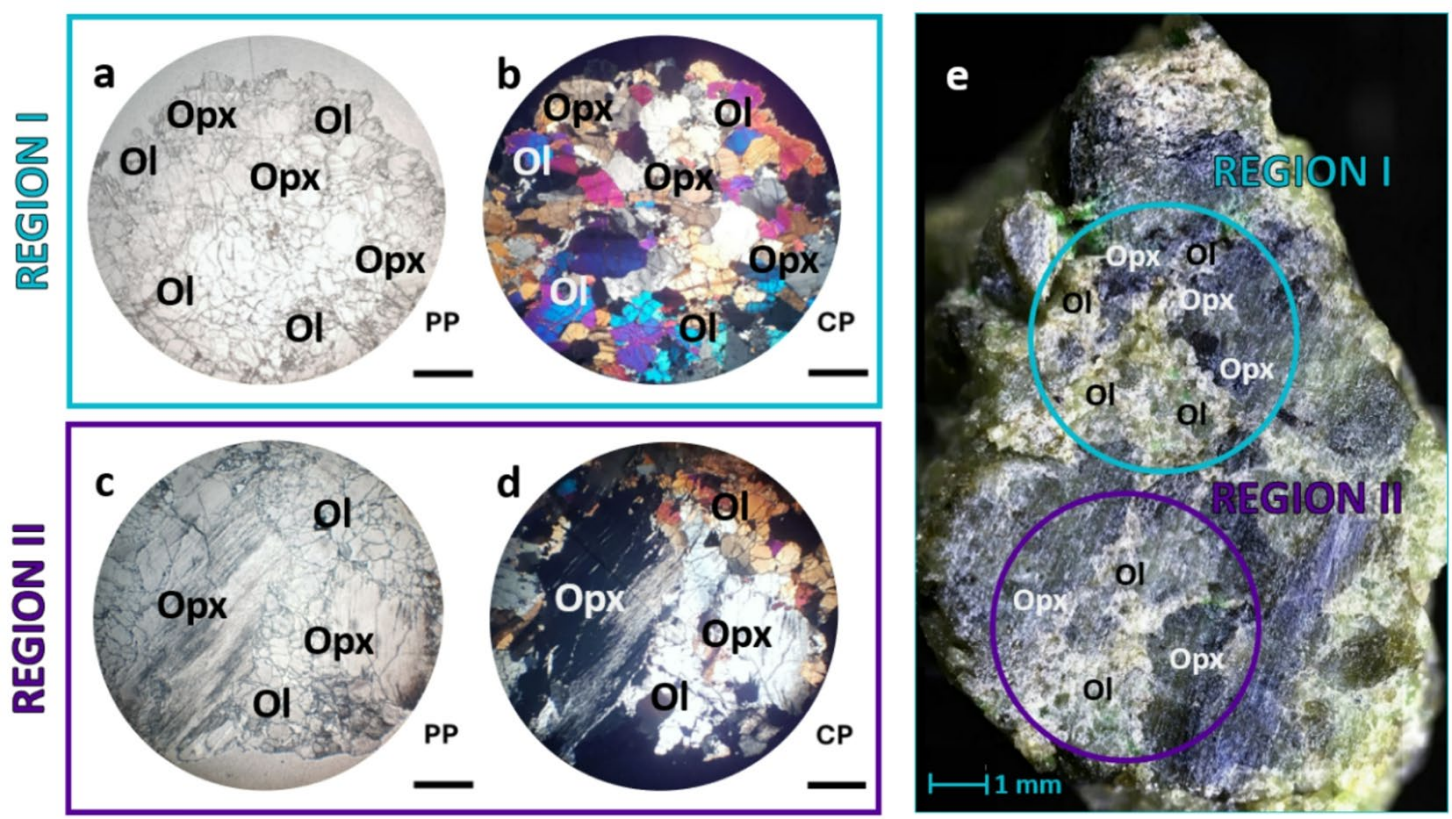
Photomicrographs of peridotite with a coarse granular microstructure of olivine and orthopyroxene. Images (**a**–**b**) highlight clean olivine crystals with high interference colors, while (**c**–**d**) show cloudy orthopyroxene aggregates. PP, plane polarized transmitted light; CP, cross-polarized transmitted light. Ol, olivine; Opx, orthopyroxene. The comparison with the original sample can be found in (**e**), where the scale bar represents 1 mm.

Peridotites were collected from the Ronda mountain range, during a sampling campaign in 2021 (Fig. 1). A sample was cut in half and polished by hand at the Department of Geological Sciences, Masaryk University (Brno, Czech Republic). Sandpaper with varying degrees of roughness (ranging from 120 to 1200 grit) was used to achieve a flat surface on the sample to ensure consistent focus in the same plane throughout the analysis. Sample characterization was carried out on a small section (14.73 × 22.75 mm) as shown in Fig. 1e.

For the petrographic study, a thin section of 30 microns in thickness was prepared from the sample and mounted onto a glass slide using epoxy resin. Observations were carried out with a Carl Zeiss petrographic microscope equipped with halogen lighting, intensity control, and light polarizers. Under the microscope, the Ronda peridotite exhibits a coarse granular microstructure. Two main mineral phases have been identified in the sections analyzed: olivine, which constitutes approximately 60%, and orthopyroxene, which makes up the remaining 40%, with no significant fraction of clinopyroxene detected in the analyzed area. According to the modal classification of ultramafic rocks based on the abundances of olivine (Ol), orthopyroxene (Opx), clinopyroxene (Cpx), as systematized by Le Maitre et al. (2002), these proportions correspond to a harzburgite-type peridotite. Although the rock’s heterogeneity may result in local variations, the observed mineralogy and texture are consistent with published descriptions of harzburgites from the Ronda massif^29^.

Olivine forms clean crystals less than a millimeter in size, with high relief (Fig. 1a) and vivid interference colors under polarized transmitted light (Fig. 1b). Orthopyroxene appears as radial fibrous aggregates up to 4 mm in size, with gray-brown colors and a cloudy appearance (Fig. 1c and d).

The process of generating a thin section from a geological sample is challenging due to the heterogeneous nature of the material. Variations in composition and texture, along with preparation steps like cutting, can introduce small discrepancies that prevent the film from fully reflecting the original sample. While this sample allows the methodology to be validated in a highly complex matrix, it should be noted that terrestrial olivines tend to exhibit higher forsterite (Mg₂SiO₄) contents than those typically observed on Mars, where more iron-rich compositions are commonly reported, reflecting lower Fo values^30^.

### Instrumentation and methodology

#### Micro–energy dispersive X-ray fluorescence

Further elemental characterization was performed using the dual M4 TORNADO µ-EDXRF spectrometer (Bruker Nano GmbH, Berlin, Germany**).** The instrument consists of a microfocus side window Rh tube powered by a low-power HV generator working at a maximum voltage/current of 50 kV/700 μA. The polycapillary optics, connected to the Rh tube, allows for microscopic measurements at spot sizes down to 25 μm, being 17 μm at 2.3 keV and 32 μm at 18.3 keV. With the aim of detecting the lightest elements, those whose atomic numbers (Z) are lower than 10, the measurements were acquired under vacuum conditions (20 mbar) using a MV 10 VARIO-B diaphragm pump. The spectrometer is also equipped with two video microscopes for the focusing process: one of them examines the sample under low magnification (1 cm^2^ area), while the other explores the final focusing (1 mm^2^ area). Finally, to generate the elemental distribution images, a deconvolution of the signals in the total spectrum was carried out, representing the whole mapped region. The distribution images are colored according to the intensity of each detected element K_α_ line. Spectra acquisition and image construction were performed using the M4 TORNADO software. The µ-EDXRF map acquisition measurement conditions included a spot size of 20 µm, a 30 µm step between measurements and an exposure of 40 ms per pixel. In addition, it should be noted that each pixel in the color maps is the average of 3 × 3 measurements.

#### Raman spectroscopy

The molecular characterization was performed using a Renishaw inVia confocal micro-Raman spectrometer (Renishaw, UK) equipped with 785 and 532 nm excitation lasers (Renishaw UK RL785 with a nominal 45 mW output power and Renishaw UK RL532C50 with a nominal 300 mW output power, respectively) and a CCD detector cooled by a Peltier device (-70 °C). The spectrometer was coupled to a Leica DMLM microscope (Bradford, UK). The equipment includes 5 × N PLAN (0.12 NA) and 20 × N PLAN EPI (0.40 NA) lenses, as well as 50 × N PLAN (0.75 NA) long-range objectives. The spot size of the laser varies depending on the laser and objective used. For the 532 nm laser, the spot size is 5.226 μm (5x), 1.568 μm (20x), and 0.836 μm (50x). For the 785 nm excitation laser, the spot size is 7.981 μm (5x), 2.394 μm (20x), and 1.277 μm (50x). The source’s nominal power can be modulated between 0.0001% and 100% of the total power to prevent sample thermo-decomposition. The inVia spectrometer is daily calibrated, setting the 520.5 cm^-1^ silicon line, and has a spectral resolution of ± 1 cm^-1^. Measurements were performed using a 5 × objective and acquisition parameters were 12 s of exposure time and 1 accumulation, while the laser power was set to 10%. Data processing involved simple and standard procedures such as baseline correction and cosmic ray removal. Mineral phase identification was performed by pre-processing data with the Wire™ 4.2 software (Renishaw) and comparing the experimental spectra with those in official databases, such as the RRUFF database (www.rruff.info), as well as in-house databases.

#### Laser-induced breakdown spectroscopy

The instrument employed for LIBS measurements was the commercial Firefly LIBS system (Lightigo, Czech Republic). The device consisted of a diode-pumped Nd:YAG solid-state laser operating at the fourth harmonic, 266 nm. The focusing UV micro-objective (13 mm focal length) allowing the spot to be reduced down to a size of 30 µm. The pulse duration and laser energy for the measurement were set to 6 ns and 10 mJ/pulse, respectively, with a repetition rate of up to 50 Hz. The analysis was carried out on a single-shot basis. Although the wavelength differs from that of SuperCam’s 1064 nm laser, and certain variations in laser-matter interaction may be expected, the detected elemental emissions remain comparable. The use of UV excitation improves spatial resolution, which is advantageous for detecting subtle compositional variations at the microscale level.

Plasma light was collected by a UV-NIR wide angle lens (37.5 mm focal length and 25.4 mm in diameter) and focused into an optical trifurcated fiber (size 200 μm**,** N.A. 0.1). The output of these optical fibers was then connected to the 25 µm entrance slit of three Czerny-Turners spectrometers fitted with a CMOS linear image sensor covering a spectral range of 190–455 nm. Detectors were set with a gate delay of 500 ns and a gate width of 50 μs. For measurements, the grating of 2400 lines mm^-1^ (blaze at UV/VIS) provides a resolution of ~ 0.035 nm**.**

This first experimental approach did not attempt to reproduce reduced atmospheres or complex gas compositions, since doing so would represent an additional instrumental challenge requiring a dedicated vacuum chamber. Instead, an argon purge was used to create a more uniform plasma and to clean the sample of dust generated during chemical mapping. Laser ablation in the presence of this particular buffer gas enhances the temperature and density gradient of the plasma. As an atomic inert gas, argon has a high atomic mass and high energy excitation levels (≥ 11.55 eV). These properties facilitate effective confinement of the vapor plume by elastic collisions, thereby reducing the energy exchange between the vapor and the gas. Correspondingly, the plasma produced in an argon environment exhibits elevated temperature and electron density, resulting in higher intensities^31^. Despite the use of an argon purge, minor contributions from residual atmospheric N_2_ may persist and affect the plasma conditions.

For the mapping process, the lateral resolution was 40 µm and the overlap between analyses was 25%. This overlapping approach contributes to minimizing blurring effects in the definition of phase interfaces, enhancing the spatial resolution when laser pulses interact with grain boundaries. The sampling depth of the analysis oscillated between ca. 5 μm and 20 μm depending on the sample topography at the actual laser shot location.

For elemental identification, a single, non-interfering emission line was selected for each element. Given the complexity of the sample, this criterion was considered the most reliable. When available, the most intense line was chosen to improve analytical sensitivity. As validation, the spatial distribution of µ-EDXRF data was compared with LIBS data to ensure the consistency of the maps.

Each LIBS spectrum from a single sampling point was normalized to the total area under its curve. Subsequently, the elemental maps were generated and normalized from 0 to 1 by scaling the intensity of each element relative to its maximum value within the entire sample. This additional normalization step was performed in order to standardize the scales across elements and to facilitate interpretation and comparison of the resulting maps.

#### Geochemical ratios in peridotite analysis

Peridotites are commonly analyzed using several elemental molar ratios to provide fundamental information on their composition and geological evolution. Interesting features are the Mg molar fraction relative to Fe (denoted as Mg#, Eq. (1)) and the Cr molar fraction relative to aluminum (denoted as Cr#, Eq. (2)). The combination of geochemical ratios of Cr# and Mg# in peridotites is an indicators for understanding the mineralogy and petrogenetic evolution of these ultramafic rocks^32,33^. Since concentrations of the elements were not available, in this work ratios based on LIBS normalized intensities (N.I.) were used. This approach not only broadens the perspective of geological analysis but also provides a fast way for the characterization of mineral phases and alteration processes, especially in contexts involving large datasets^34^.

$$Mg\#= \frac{ N.I. \left(Mg I 280.27 nm \right)}{N.I.\left[\left( Mg I 280.27 nm\right)+\left(Fe I 404.58 nm\right)\right]} (1)$$

$$Cr\#= \frac{N.I. \left(Cr I 425.43 nm\right)}{N.I. \left[\left(Cr I 425.43 nm\right)+\left(Al I 309.27 nm\right)\right]} (2)$$

#### K-means method

The k-means method is a powerful tool of unsupervised learning methodologies. It iteratively assigns points to clusters and recalculates centroids until convergence is achieved. k-means clustering aims at partitioning a dataset into clusters (k) based on data point similarity, which highlights its robustness in identifying underlying patterns without relying on predefined labels. The k-means methodology implemented in MATLAB utilized a variation of the Lloyd algorithm, it seeks to minimize the sum of squared distances within each cluster. This process involves assigning each data point to the cluster whose nearest centroid minimizes the Euclidean distance. Mathematically, the k-means algorithm for a dataset X and k clusters, can be expressed by minimizing the cost function Eq. (3)^35,36^.

$$J \left(C\right)= \frac{1}{M}{\sum }_{j=1}^{k}{\sum }_{i=1}^{M}\Vert {x}^{(i)}{{-\mu }_{j}\Vert }^{2} (3)$$

where J represents the sum of squared Euclidean distances between data points and their assigned cluster centroids. M signifies the number of data points in the dataset, while k denotes the number of clusters or centroids. The variables χ (i) and µ _j_ represent data point and the centroid of the cluster, respectively.

In that case, the k-means algorithm divides the spectral data into clusters, representing the various expected mineral components within the rock samples. Each data point is assigned to the cluster whose centroid is closest, which is equivalent to identifying the ‘chemical similarity’ between mapping points. This similarity can be understood as the comparative analysis of spectral profiles within the feature space, reflecting the presence and abundance of the elements identified by LIBS.

The selection of the optimal number of clusters (k) ensures the validity and significance of the results. In this sense, a systematic approach involving silhouette and elbow analysis was employed, assessing the cohesion and separation of mineral groups, and providing an objective criterion for identifying the most suitable number of clusters. The procedure is described in the supplementary material.

Although multivariate analyses often involve larger input matrices, k-means clustering was applied to a selected set of spectral lines from LIBS maps (see **Table S1**). This approach reduces dimensionality while retaining key chemically relevant information, making the data easier to interpret and more computationally efficient. Similar methods have been successfully used in previous studies, where discrete spectral lines were employed for clustering, demonstrating the effectiveness of this approach in spectral analysis^37–39^.

## Results and discussion

### Elemental characterization

Elemental analysis of peridotite constituted the first step of our method and was performed using LIBS and μ-EDXRF. LIBS is particularly suited for the analysis of light elements with low ionization energy, whereas μ-EDXRF is more sensitive for elements with larger atomic numbers. In both instances, Al, Ca, Cr, Cu, Fe, Mg, Mn, Ni and Si were identified. Additional trace elements distinguished by μ-EDXRF were Ti, V and Zn. Elements detected by LIBS not seen by μ-EDXRF include Sr, Na, Ba, along with C in both atomic and molecular form. Table S2, in the supplementary material, summarizes the list of elements detected by LIBS and μ-EDXRF.

**Fig. 2:**
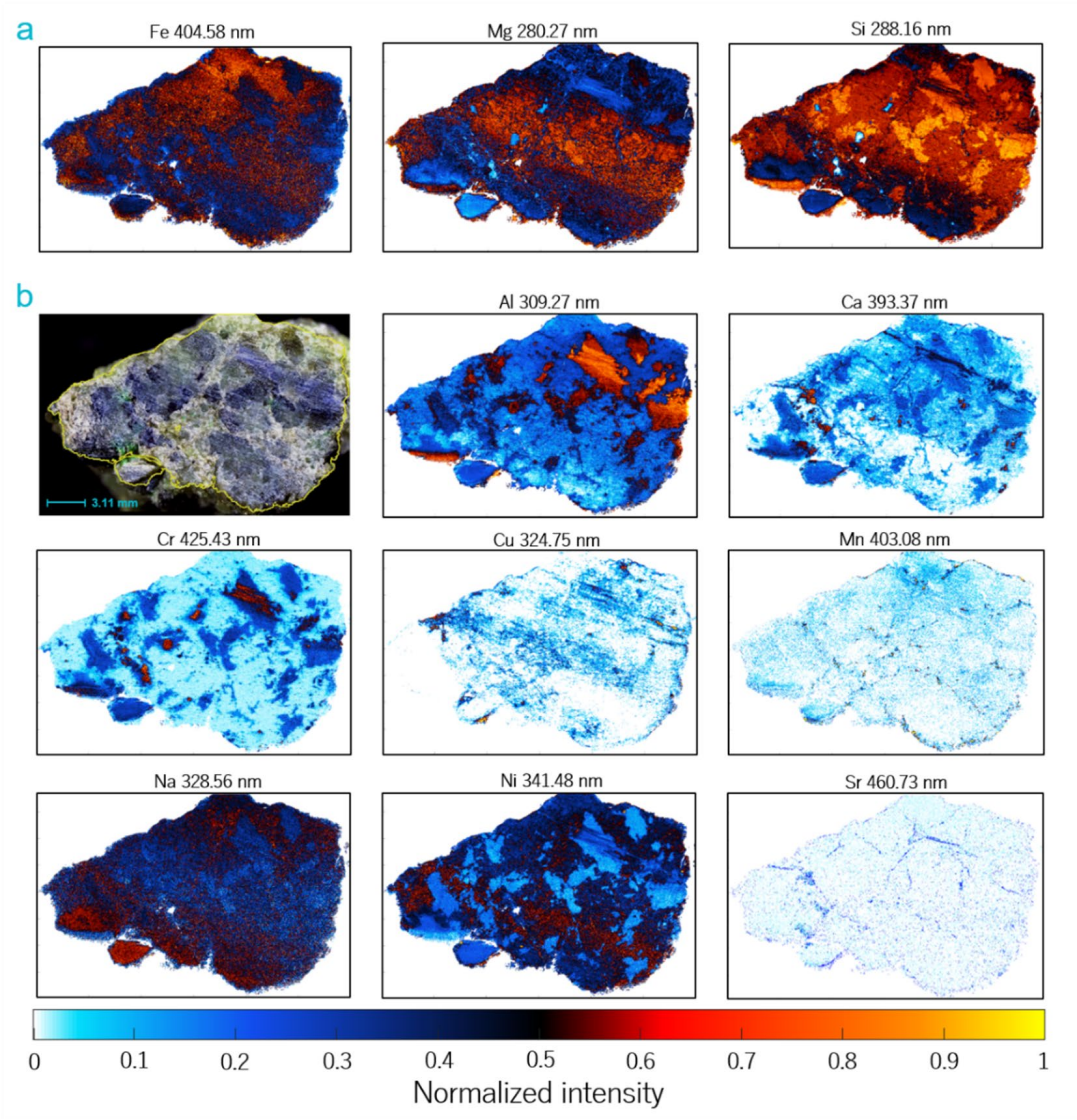
(**a**) LIBS maps of the main elements in the rock acquired at specific wavelengths. (**b**) LIBS maps of minor elements including Al, Ca, Cr, Cu, Mn, Na, Ni, and Sr in a sampling area of 14.73 mm × 22.75 mm.

Based on the LIBS and μ-EDXRF spectral data, two-dimensional maps were acquired according to the mathematical algorithm described in the experimental section. Figure 2a shows the spatial distribution of the main species observed by LIBS. Wavelengths of emission lines associated with each element are also indicated. The color scale shown in the images serves as an indicator of elemental abundance. However, it is important to emphasize that the normalized intensity of specific spectral lines may be influenced by the properties inherent to distinct mineralogical phases within the sample^40^. Even after normalization, the exhibited coloration on the maps may convey information about these intrinsic physical characteristics (hardness, color, absorption, among others). In the maps, white color denotes the absence of the element. Figure S1 shows the distribution of the major elements along the sample measurement by μ-EDXRF. The maps obtained using both techniques show agreement in the distribution of the monitored common species such as Fe and Si^41^.

Chemical maps of minor elements are shown in Fig. 2b. Except for Cu, Mn, and Sr, which are absent in broad areas of the rock, Al, Ca, Cr, Na, and Ni can be found in most of the locations inspected. Across the area under study a fair correlation between Al and Cr is observed. Locations showing the highest Al and Cr intensity (red clustered in the central region of the slice) match with the distribution of mineral phases of the sample surface, which could be compatible with the presence of amphiboles, pyroxenes, or spinels^42,43^. Calcium is located predominantly in small beta phases and veins crossing the sample. Strontium, involved in ion-exchange hydrological processes in soils and sediments, has been detected forming various veins matching the Ca-rich zones. This finding could serve as an indicator of geological processes occurring during serpentinization, where Sr may replace Ca in minerals or silicates like dolomite (CaMg(CO_3_)_2_) or tremolite (Ca_2_Mg_5_Si_8_O_22_(OH)_2_), a phenomenon influenced by hydrogeological conditions and soil characteristics^44,45^.

The spatial distribution of Mn reveals veins mainly along fractures and at contacts between domains of different coloration observable in the sample image. These Mn-enriched zones are concentrated at the margins delimiting optically contrasting regions, in particular between areas of dark blue-green shades and lighter sectors, as well as in some peripheral zones as observed in Fig. 2b (more details in Fig. S2)^46,47^. The presence of Na in the sample is ubiquitous, although some regions of the lower section appear to be slightly enriched. Furthermore, its distribution seems to coincide with areas of magnesium depletion.

Nickel is present throughout the sample probably due to its initial formation by magmatic processes in which olivine, the major mineral of peridotites, incorporates Ni as it crystallizes. Over time, hydrothermal, weathering, and metamorphic processes contribute to the persistence of Ni in these ultramafic rocks^48^.

**Fig. 3:**
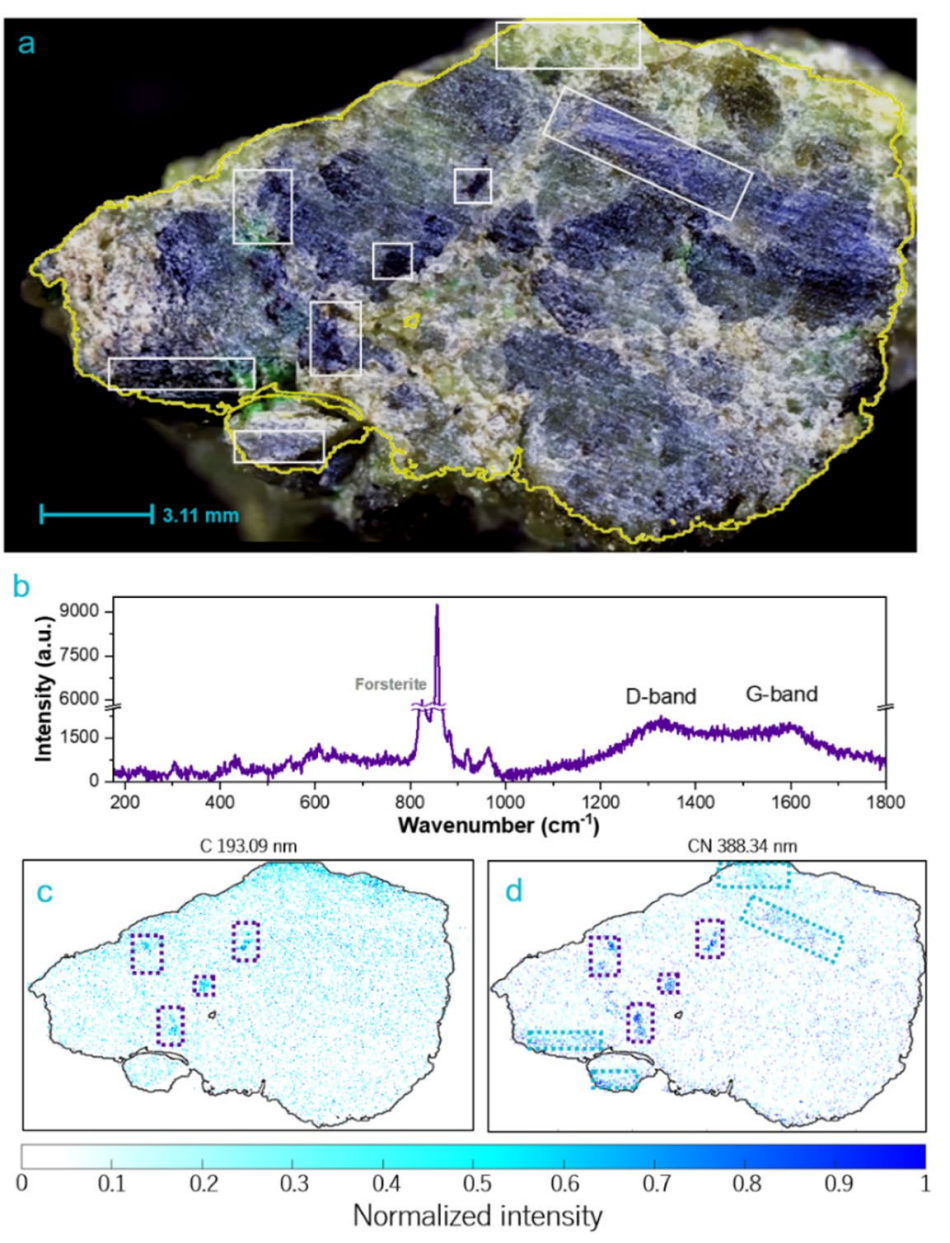
(**a**) Detailed view of the carbon-rich area, sampling dimension 14.73 mm × 22.75mm, (**b**) Raman spectrum of carbon rich area in the sample. Measurement conditions: 785 nm, objective 5x, 12 s exposure time, 1 accumulation and 10% laser. (**c**) and (**d**) show the LIBS intensity of C I at 193.09 nm and CN at 388.34 nm, respectively. The purple and blue boxes indicates the carbon distribution identified for both recorded atomic and molecular band.

The sample also revealed areas with a significant abundance of carbon, presumably attributed to carbonate phases and/or to the presence of organic matter. Figure 3 depicts the distribution of two species: atomic carbon studied at 193.09 nm and the molecular CN violet system at 388.34 nm, corresponding to the Δν = 0 vibrational mode of the (0,0) transition. It should be noted that the composition and pressure of the background atmosphere may influence the formation and relative intensity of these species, as reported in the literature^49,50^.

Some regions show a less dense scatter of dots of low intensity, with an appearance that evokes veins or seepage, especially noticeable in areas close to fluids. Significantly, a correlation is observed with a higher Ca abundance in the same areas. This pattern suggests the presence of alteration minerals, such as carbonates, or the influence of possible serpentinization processes^51^. This phenomenon can be accelerated by the presence of Al^52^, which is consistent with the chemical maps shown in Fig. 2b, where Al is mainly distributed in areas where signals due to carbon appear. The correlation between Cr and Al with C in peridotites has been previously documented, describing the association of graphite or diamonds with chromites, olivines, and sulfides, which is consistent with the observed results^53–55^.

The carbon signal is notably stronger in the CN 388.34 nm feature, which aids in identifying areas with medium and low carbon abundances. Chromatic variations in the sample are observed in regions associated with carbon, particularly in areas located in the central region of the sample (highlighted in purple). These sections are noticed as the darkest regions of the sample as observed in Fig. 3a, and appear to be associated with areas rich in chromium.

Raman spectroscopy helps in revealing the nature of the carbon signatures identified by LIBS. The detection of characteristic Raman bands G and D provides complementary evidence for the presence of carbonaceous material within the sample, as shown in Fig. 3b. These bands, observed at 1580 cm^-1^ (G band) and 1350 cm⁻^1^ (D band), are indicative of graphitic and disordered carbon structures. These bands were found to be associated with minerals such as enstatite and forsterite. This association may be the result of metasomatism, hydrothermalism, or interaction with carbon-rich fluids in the geological environment in which the peridotites were formed. The identification of these carbon-bearing minerals deepens our understanding of carbon sequestration processes in ultramafic environments and highlights their significance in global carbon cycling dynamics, with similar associations of reduced carbon and primary silicates also documented in Martian meteorites^56^.

### LIBS and Raman spectroscopy for mineral characterization

**Fig. 4:**
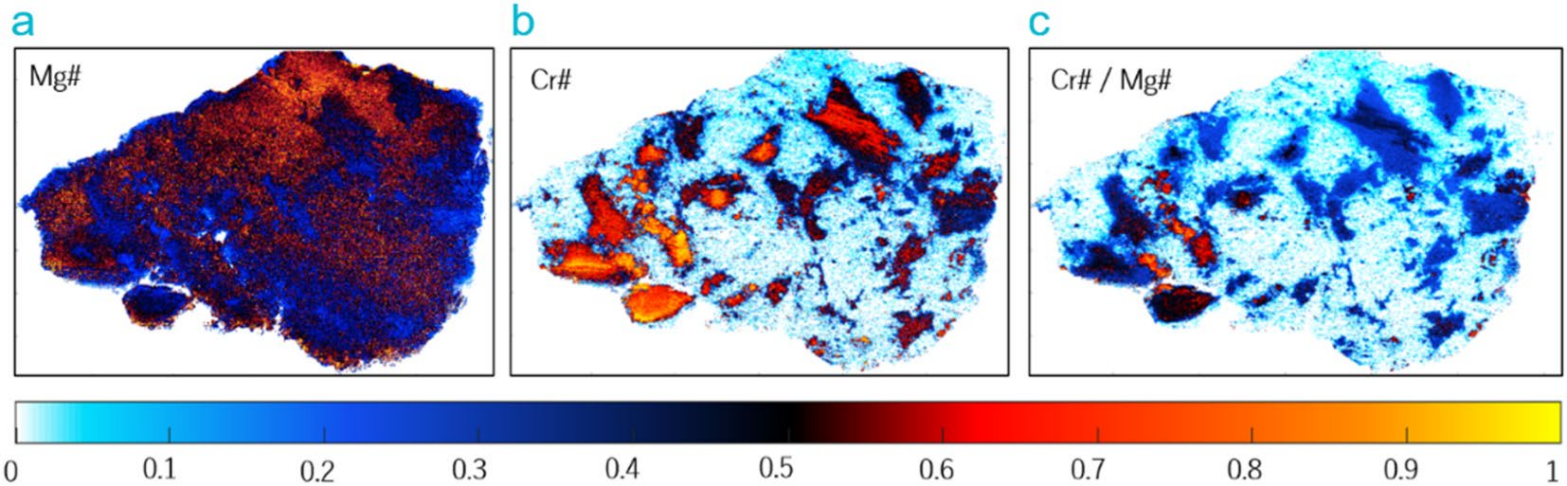
Spatial distribution of intensity ratios of (**a**) Mg relative to Fe or Mg#, (**b**) Cr relative to Al or Cr#, (**c**) Cr# / Mg# highlighting variations in chromium content relative to magnesium across the mapped region.

Figure 4 shows the LIBS intensity ratio distributions across the sample. As shown in Fig. 4a, extended zones with an elevated Mg fraction relative to Fe (Mg#) appear in the upper section of the sample, suggesting the presence of forsterite. The Cr fraction relative to Al (Cr#) is associated with the presence of Cr-rich minerals^57^. In Fig. 4b, results indicate a large Cr fraction in the left, lower part of the sample. A high Cr ratio combined with a low Mg ratio suggests the presence of chromiferous minerals like chromite or spinels mixed with other phases, which may indicate the influence of specific geological environments such as ultramafic magmatism associated with subduction zones^57,58^. This relationship may be particularly useful for identifying Cr-rich domains, such as chromite-bearing zones, which are difficult to detect on Mars due to spectral limitations and the absence of current direct mineralogical confirmation^3^. This situation may be related to partial melting processes in the Earth’s mantle, resulting in the creation of peridotites enriched with chromite. On the other hand, a low Cr ratio and a high Mg ratio would primarily indicate the existence of mafic minerals, such as olivine, as mentioned above, suggesting more extensive partial melting conditions in the mantle. As seen in Fig. 4c, olivine seems to be profusely represented across the sample. Intermediate values of Mg# and Cr# may indicate a mixture of mineral phases such as spinel and pyroxene, possibly related to metamorphic or alteration processes.

With the aid of micro-Raman spectroscopy, further phase characterization was performed. Raman analysis determined that olivine and pyroxenes were the main mineral phases that conformed the matrix. According to Torre-Fdez et al*.*^59^, olivine is a mineral composed of two endmembers: forsterite (Fo, Mg_2_SiO_4_), as the Mg endmember, and fayalite (Fa, Fe_2_SiO_4_), as the Fe one. Both endmembers of olivine have their main Raman bands as a doublet in the 810–860 cm^-1^ spectral range: 824 and 857 cm^-1^ for the forsterite and 814 and 840 cm^-1^ for fayalite.

**Fig. 5:**
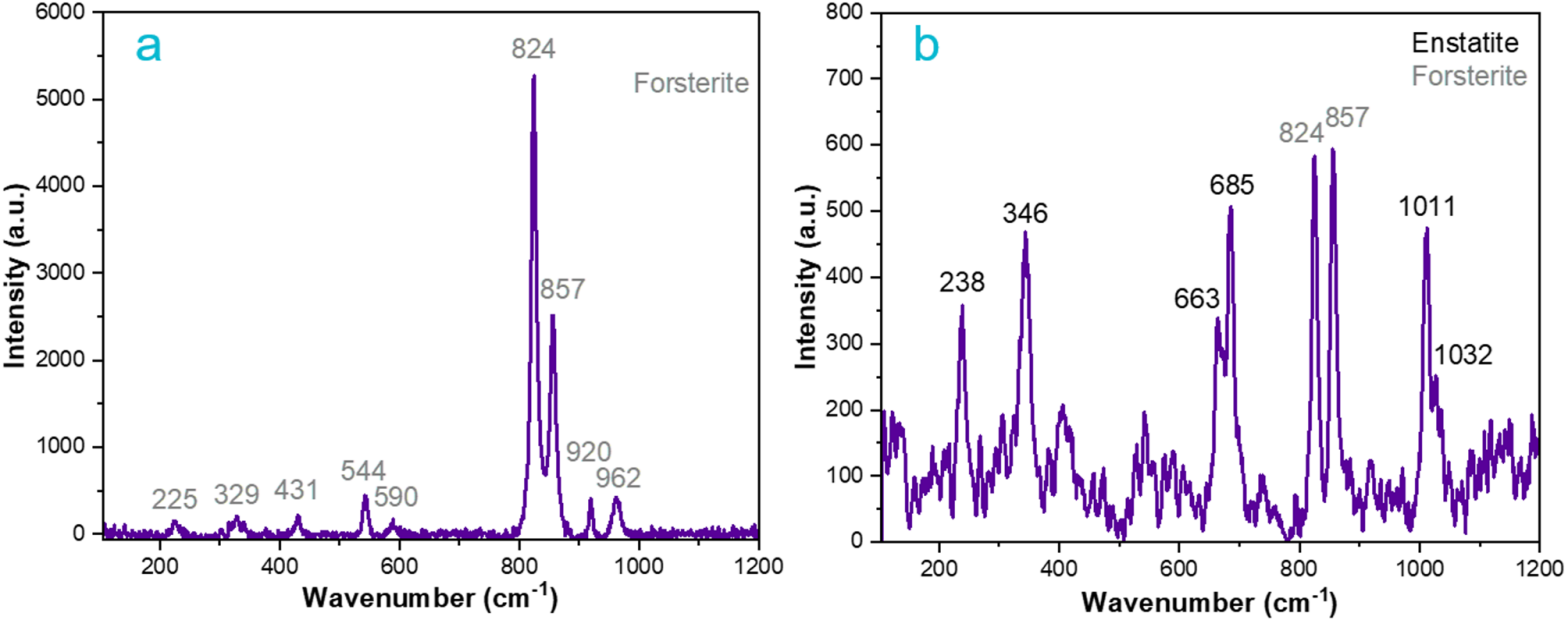
(**a**) Raman spectrum of forsterite on the sample. (**b**) Raman spectrum of a section of the sample where enstatite and forsterite coexist in the peridotite sample, peak labels in black corresponds to enstatite and gray to forsterite. Measurement conditions: 785 nm, objective 50x, 12 s exposure time, 1 accumulation and 10% laser power.

Figure 5a shows an example of the Raman spectrum of forsterite found across the sample. Raman bands appear at 962 (medium, m), 920 (m), 857 (very strong, vs), 824 (vs), 590 (weak, w), 544 (m), 431 (w), 329 (w) and 225 (w) cm^-1^. Moreover, the different fayalite (Fa, 100% Fe olivine) / forsterite (Fo, 100% Mg) olivine phases present in the peridotite were obtained using a developed Raman methodology, corresponding to forsterite with 93.8% Mg in all measurements conducted^60^.

In addition, as a consequence of the serpentinization of the peridotite, pyroxenes were identified as primary phases in the sample. In this regard, the pyroxene group is the most important mineral group in terms of the information recorded about petrogenetic processes^59^. As described by Poblacion et al*.* (2023)^61^, the spectrum of pyroxenes is divided into five Raman regions: the R1 between 800 and 1100 cm^-1^ with a strong band near 1000 cm^-1^, R2 between 600 and 800 cm^-1^ region with a strong doublet peak or an asymmetric single band near 670 cm^-1^, R3 between 300 and 450 cm^-1^ with a group of strong overlapping bands, R4 between 450 and 600 cm^-1^ with a group of medium overlapping bands and R5 below 300 cm^-1^ with or without a few peaks of moderate intensities.

Figure 5b shows the Raman spectrum of a type of pyroxene with a doublet in the R2 region between 600 and 800 cm^-1^ and a doublet in the R3 region between 300 and 450 cm^-1^, that correspond to a high Mg *Pbca* orthorhombic space group mineral known as enstatite (Mg_2_Si_2_O_6_)^60^, whose Raman bands appeared at 1032 (medium), 1011 (strong, s), 685 (s), 663 (s), 346 (s) and 238 (s) cm^-1^. Thus, as described by Huang et al*.*^62^, the Raman active vibrational modes obtained in the Raman spectrum correspond to a pyroxene En_.975_Fs_.025_, or a composition of 97.5% enstatite (MgSiO_3_) and 2.5% ferrosilite (FeSiO_3_).

**Fig. 6:**
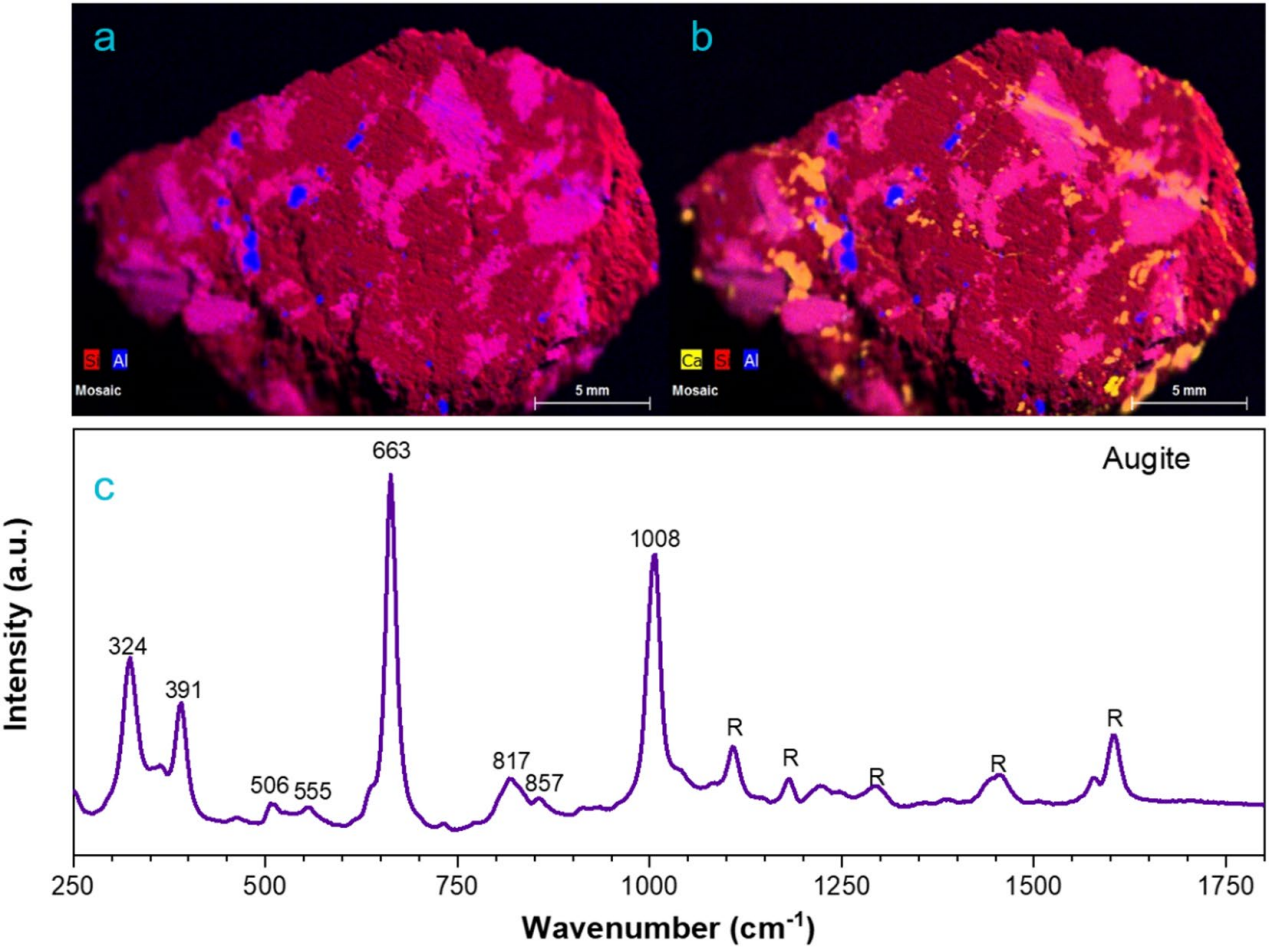
Optical superposition images of the elemental distribution for (**a**) Al and Si and (**b**) Al, Ca, and Si. These micro–energy dispersive X-ray fluorescence elemental maps were acquired at 50 ms and 1 cycle/frame counts. (**c**) Raman spectrum from augite on the sample.

The potential presence of calcium-rich pyroxenes has also been identified through micro–energy dispersive X-ray fluorescence analysis. Figure 6a illustrates the distribution of silicon (in red) and aluminium (in blue) throughout the sample, which are essential elements in the chemical composition of pyroxenes. Upon closer examination, the areas coloured in pink indicate the coexistence of both elements. The addition to calcium (yellow), Fig. 6b reveals the coexistence of calcium, silicon and aluminium in certain orange-colored areas, which may indicate the presence of calcium-rich pyroxenes such as augite.

The presence of augite is further confirmed by Raman spectroscopy. Figure 6c shows a typical spectrum of this calcium rich pyroxene with bands appearing at 1008 cm^-1^, 663 cm^-1^ and a characteristic doublet at 324 cm^-1^ and 391 cm^-1^. In contrast to the Raman spectra of enstatite, in this case the intensity of the R2 band (663 cm^-1^) is larger than the one at R1 (1008 cm^-1^), which is due to the presence of Ca in the structure as shown in previous studies by Huang et al*.* and Buzatu et al*.*^62,63^. The presence of a doublet in the low frequency region (R3) also indicates that this augite has a lower content in Fe.

### Mineral assemblages within the peridotite sample

Apart from the major phases described above, the large diversity of major and minor elements observed by LIBS suggests the existence of a considerable variety of additional minerals in the peridotite sample. In an attempt to find their distribution, the k-means method was applied to LIBS data. The k-means algorithm is known for its adaptability to a variety of data, demonstrating successful results in LIBS and Raman image analysis^64–66^.

**Fig. 7:**
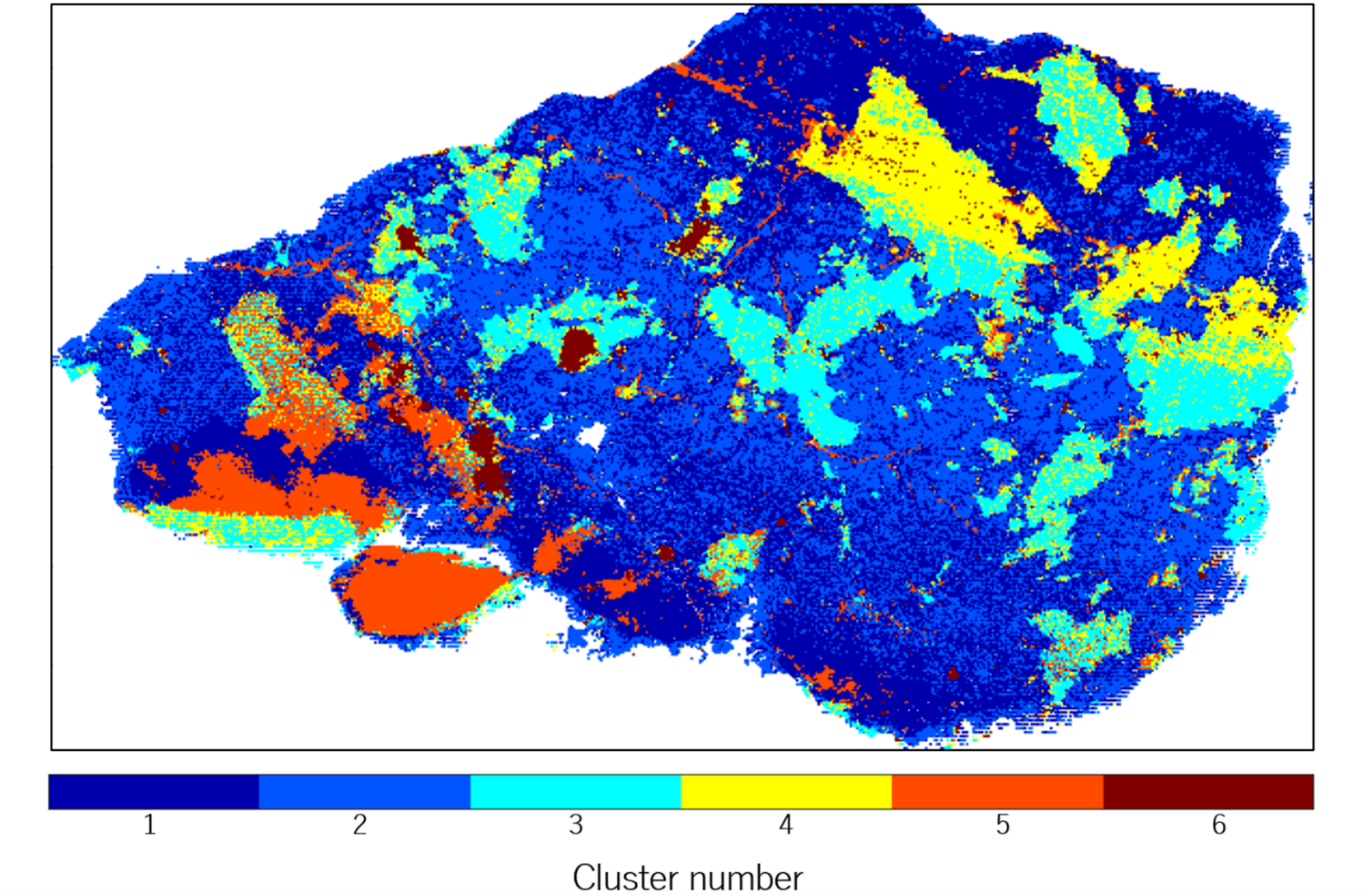
Spatial distribution of k-means clusters along the sample. Elements that had the highest impact on the classification were Fe, Mg, and Na.

The k-means algorithm was applied to a data matrix composed of the normalized LIBS intensity of the 11 elements identified in each sampling point (see Table S1). These normalized intensities were obtained by dividing the intensity value by the total sum of the spectrum. The results of this analysis are presented in Fig. 7, where the color code represents the clusters or groups identified. In this case, six distinct groups were obtained. The centroid of each cluster corresponds to the average normalized intensity of the chemical elements within that cluster. A detailed summary of the centroids is provided in the supplementary material (see Table S3). This methodological approach guarantees a consistent and uniform representation of the spectral data, enabling meaningful comparisons across all sampled points within the peridotite. As shown, 6 regions with similar spectral characteristics, which may correspond to different mineral phases or association of some mineral phases could be assigned. Upon observing the generated clusters map and considering the nature of the sample, it is noticed that there is a wide mixture of phases present. The clusters were subsequently interpreted as mineral phases based on two main criteria: (1) the average of the normalized intensity of the chemical elements within each cluster (detailed in Supplementary Table S3), and (2) the mineralogical assignments confirmed through micro-Raman spectroscopy conducted on representative points of each group. These Raman identifications served as ground truth for validating the chemical classification. Additionally, the use of elemental ratios such as Mg# and Cr# (as shown in Fig. 4.) supported and confirmed the interpretation of certain domains.

The associations between the different clusters obtained were observed. In the case of clusters 1 and 2, they appeared to represent the matrix of the sample and the predominant phases. These data were validated with Raman spectroscopy and petrographic analysis. The group of data in cluster 1 has a higher abundance of Fe and Mg, and their normalized value is closer to 0.49 and 0.34, respectively. The high abundance of Fe and Mg, together with moderate levels of Al and Ca suggested the presence of enstatite rich in Fe (orthopyroxene), potentially associated with forsterite (olivine). Cluster 2 exhibited a composition similar to that of group 1, but in this instance, it exhibited a larger abundance of Mg, which could correspond to the other main phase of the sample, forsterite-rich olivine^67^.

The spatial distribution of cluster 3 is clearly delineated within the central region of the sample. It is characterized by a pronounced enrichment in Mg and Si, in addition to a notable presence of Fe, Al, and Ca. These elemental distributions were also obtained by µ-XRF data. This composition suggests a probable admixture of orthopyroxenes. This interpretation is consistent with the data obtained from petrographic and Raman analysis and aligns with bibliographic data for harzburgite-type peridotites^67^.

Cluster 5 manifests as veins running along the sample and more delimited spatial areas in the lower left region. The presence of elevated levels of Ca and Na observed in the sample indicates that these phases may have resulted from contact with hyperalkaline fluids and represent alteration minerals. This observation suggests that metasomatic processes may have occurred.

Group 6 is of particular interest as it corresponds to carbon-rich regions. This cluster exhibits a high proportion of Cr and Fe, which may indicate the potential presence of spinels or oxides, such as chromite, and other associated minerals. Points within this cluster have been corroborated through Raman spectroscopy, confirming this mineralogical composition.

Full interpretation of this sample is, though, challenging and further analysis is required for a more precise understanding of these associations. Nonetheless, complementary techniques have successfully validated some of our assumptions. Therefore, cluster analysis can be a useful tool for fast and efficient determining the number of phases, their distribution, and potential interrelationships among mineral phases and oxides in rocks.

## Conclusions

In this study, an integrated methodological approach was applied to describe the compositional and mineral chemistry of a peridotite sample collected from the outcrop in Ronda. The combined use of LIBS, micro-EDXRF and Raman spectroscopy, permits a detailed and concordant understanding of the mineral phases constituting the sample. The combined data reveal a complex mineralogy dominated by olivine, orthopyroxene, with each technique confirming and complementing the findings of the others. The approach also proved effective in detecting light elements and carbon-bearing species. LIBS identified atomic and molecular carbon emissions, while Raman spectroscopy confirmed their presence through the characteristic D and G bands. This integration enabled the localization of carbon-rich domains associated with phases such as orthopyroxene or chromite, illustrating their potential for addressing key geochemical questions in planetary exploration.

Elemental ratios such as Cr# and Mg# derived from LIBS data, have proven to be effective in identifying Cr-rich domains, offering a practical alternative for the indirect detection of spinels and chromite under analytical conditions where their direct identification remains challenging. Combined with automated classification using k-means clustering, this strategy enables rapid geochemical segmentation of complex lithologies. This strategy is particularly well suited for planetary missions, where operational limitations often restrict the number of detailed point analyses. Moreover, its ability to efficiently organize and interpret large spectral datasets makes it especially valuable for extended missions, where data accumulation over time can hinder analysis. Overall, the comprehensive approach used here provides a robust framework for understanding the petrological and geochemical characteristics of samples of complex mineralogy as is the case of the peridotite rock from the Ronda massif.

## Supplementary Information

Below is the link to the electronic supplementary material.

Supplementary Material 1

## Data Availability

The datasets used and/or analyzed during the current study are available from the corresponding author upon request.
