# Supplementary material for: Integration of SuperCam based chemical imaging and clustering to correlate geochemistry and mineralogy in heterogeneous samples
Source: Sci Rep. 2025 Oct 29;15:37808. doi: 10.1038/s41598-025-21770-4 (PMC12572376; doi:10.1038/s41598-025-21770-4)
Supplement: Supplementary file 1 — Supplementary Material 1 [file 41598_2025_21770_MOESM1_ESM.docx]

Supplementary

**Table S1.** Elements selected and their wavelength for the K-means analysis.

| Species | Wavelength (nm) |
| --- | --- |
| Al | 309.27 |
| Ca | 393.37 |
| CN | 388.33 |
| Cr | 425.43 |
| Fe | 404.58 |
| Mg | 280.27 |
| Mn | 403.08 |
| Na | 328.56 |
| Ni | 341.48 |
| Si | 288.16 |
| Sr | 460.73 |

A comparison of elemental constituents found in the Ronda peridotite using both LIBS and µ-EDXRF is shown in the Table S2.

**Table S2.** Elements found in the Ronda peridotite sample by LIBS and μ-EDXRF.

|  |  | | **Elements** | | | | | | | | | | | | | | |
| --- | --- | --- | --- | --- | --- | --- | --- | --- | --- | --- | --- | --- | --- | --- | --- | --- | --- |
| **Technique** | **Al** | **Ba** | | **C** | **Ca** | **Cr** | **Cu** | **Fe** | **Mg** | **Mn** | **Na** | **Ni** | **Si** | **Sr** | **Ti** | **V** | **Zn** |
| **LIBS** | **✓** | **✓** | | **✓** | **✓** | **✓** | **✓** | **✓** | **✓** | **✓** | **✓** | **✓** | **✓** | **✓** | - | - | - |
| μ-EDXRF | **✓** | - | | - | **✓** | **✓** | **✓** | **✓** | **✓** | **✓** | **-** | **✓** | **✓** | - | **✓** | **✓** | **✓** |

In the figure S1, it is shown the relative presence of the major elements along the sample. The colour intensity of the images is directly correlated with the concentration of the elements, the higher the colour intensity, the higher the concentration. On the contrary, the black areas represent the lack of the elements. In this sense, the relative presence of the major elements ordered from the highest to lowest presence was Mg, Si, Fe, Ca and Ni. In terms of the distribution of the elements, it can be seen in the Figure 1, Si was present in the empty spaces of Ni, Mg and Fe. On the other hand, the distribution of Ca did not coincide with the distribution of the other elements.

**
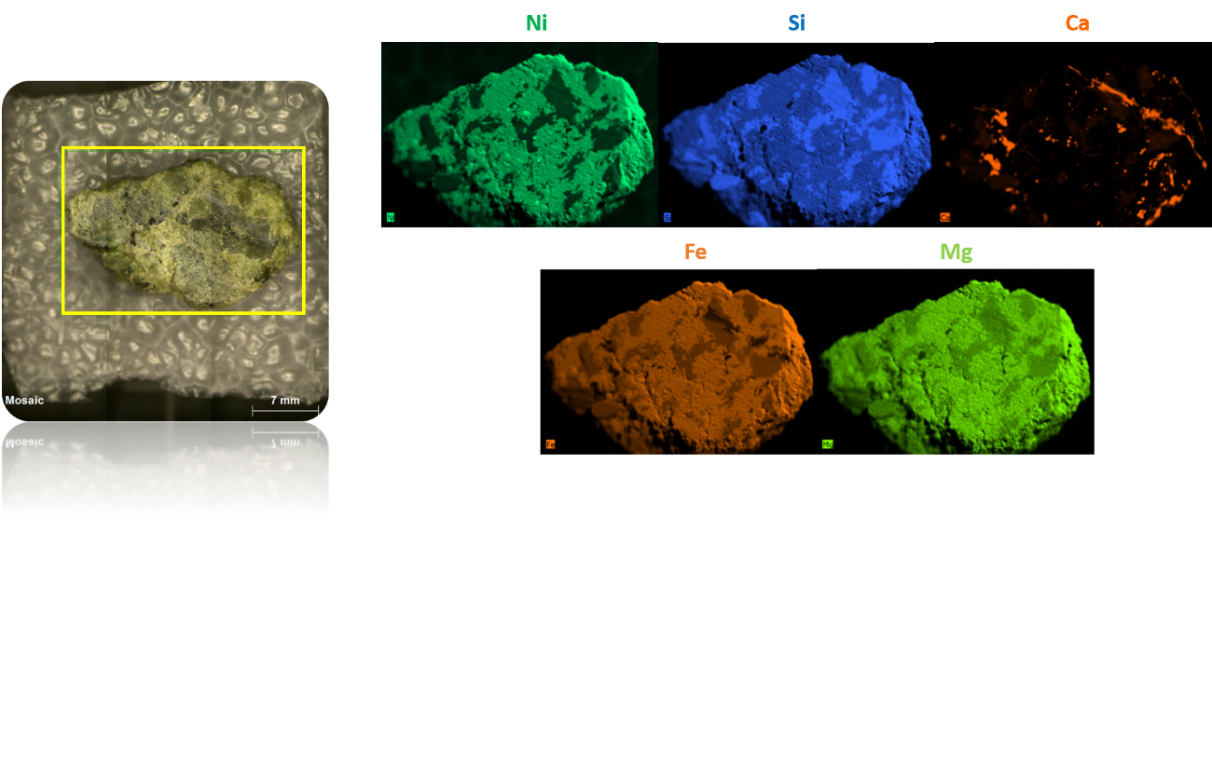
**

**Figure S1.** The optical image of the μ-EDXRF mapped area and the elemental distribution for Ni, Si, Ca, Fe and Mg. These elemental maps were performed at 50 ms and 1 cycle/frame counts.


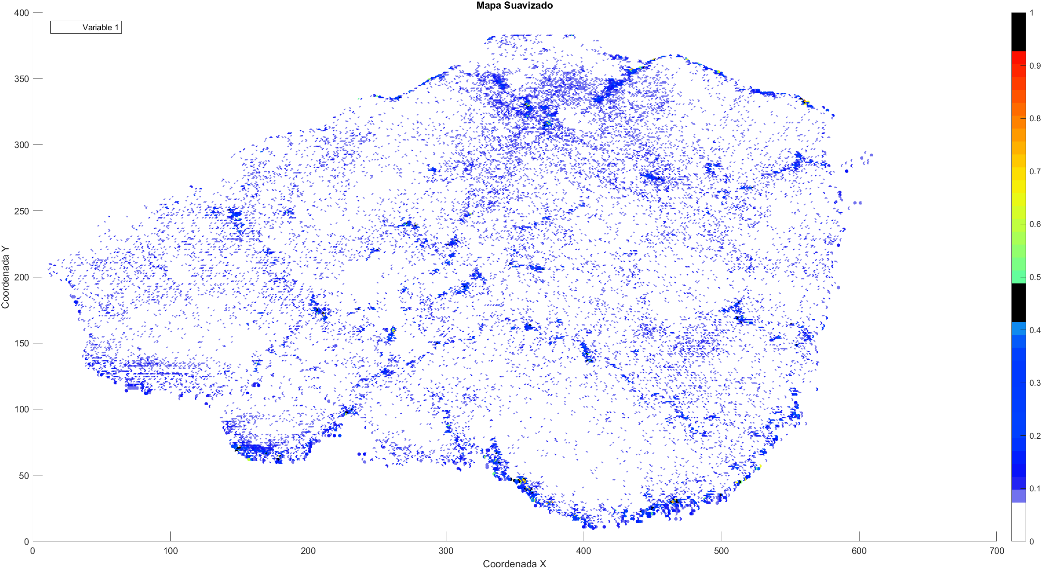


**Figure S2.** Manganese vein distribution in the analyzed peridotite sample.

The elbow method was utilized to graphically represent the relationship between the number of clusters and the model's inertia, where inertia (I) denotes the dispersion of data points within each cluster, equation (4). The resulting plot highlights an inflection point, or 'elbow,' offering a potential guide for optimal cluster count determination [1, 2]

$$I \left( k \right)=\sum_{i=1}^{n} {min}_{\mu_{j}}\in C\left( \left\| x_{i}-\left. \mu_{J} \right\|^{2} \right. \right) (4)$$

Where, n denotes the total number of data points, χ_i_ represents data point, μj signifies the centroid of the j-cluster, C denotes the set of clusters, and ∣∣⋅∣∣ denotes the Euclidean norm. The resultant plot highlights an inflection point, or 'elbow,' providing a potential guide for optimal cluster count determination. (SEE) WCSS

On the other hand, silhouette analysis evaluates the quality of clustering by assessing both cohesion and separation of clusters based on individual data point distances, as shown in equation (5) [3].

$$s\left( i \right)= \frac{b\left( i \right)-a(i)}{max\left\{ a\left( i \right),\left. b(i) \right\} \right.} (5)$$

In this context, a(i) denote the average distance from the point to other points within the same cluster, while b(i) represents the minimum average distance from the point to points in a different cluster. The silhouette score ranges from -1 to 1, with higher scores indicating better clustering quality. A plot of silhouette scores for varying numbers of clusters may help identify the optimal number of clusters by selecting the peak score. However, it is important to recognize the complexity of data clustering, as demonstrated by the occasional presence of multiple inflection points in the elbow method and varying silhouette scores. This indicates the need for careful examination and interpretation when determining the appropriate clustering configuration.


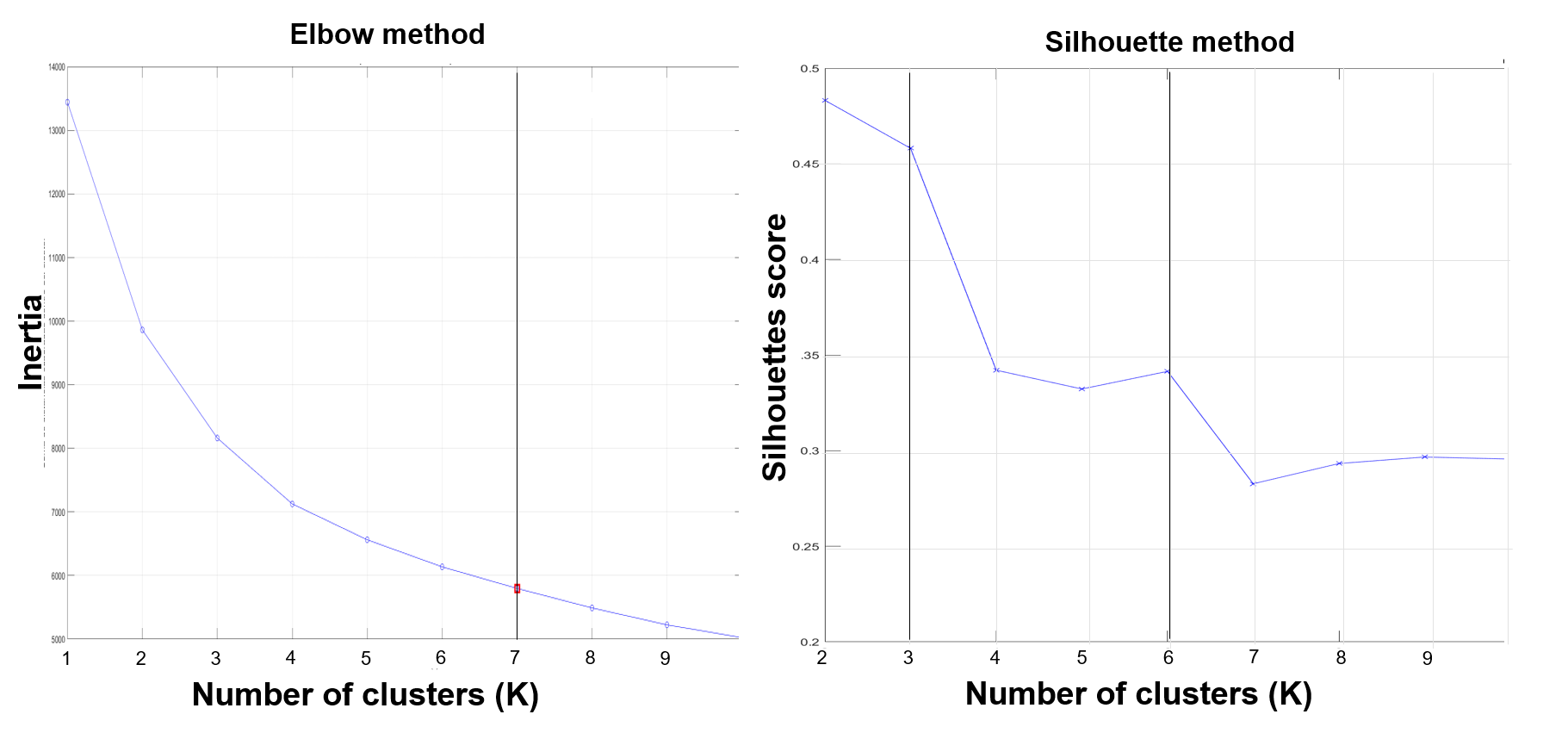


**Figure S3.** On the right, the k-means curve of the elbow method; on the left, the plot of the silhouette method.

**Table S3**. K-means centroids, which are the average of the normalized intensity of the points within each cluster for 11 selected elements.

|  | **Species** | | | | | | | | | | |
| --- | --- | --- | --- | --- | --- | --- | --- | --- | --- | --- | --- |
| **Cluster #** | **Al** | **Ca** | **CN** | **Cr** | **Fe** | **Mg** | **Mn** | **Na** | **Ni** | **Si** | **Sr** |
| **1** | 0.19 | 0.14 | 0.03 | 0.02 | 0.45 | 0.34 | 0.05 | 0.47 | 0.26 | 0.43 | 0.00 |
| **2** | 0.16 | 0.13 | 0.02 | 0.01 | 0.39 | 0.51 | 0.03 | 0.34 | 0.22 | 0.52 | 0.00 |
| **3** | 0.35 | 0.27 | 0.02 | 0.15 | 0.35 | 0.52 | 0.03 | 0.32 | 0.12 | 0.66 | 0.00 |
| **4** | 0.44 | 0.30 | 0.04 | 0.27 | 0.42 | 0.34 | 0.06 | 0.37 | 0.16 | 0.53 | 0.01 |
| **5** | 0.22 | 0.35 | 0.04 | 0.17 | 0.39 | 0.27 | 0.06 | 0.46 | 0.17 | 0.40 | 0.01 |
| **6** | 0.38 | 0.13 | 0.08 | 0.44 | 0.41 | 0.18 | 0.05 | 0.40 | 0.17 | 0.22 | 0.01 |

**Consortium**

**SIGUE-Mars team**

D. Carrizo^8^, J. M. Madariaga², J. Laserna^1,3^, M. Taravillo^6^, J. Martínez-Frías^9^, T. Belenguer^11^, S. H. Faria^12^, E. Mateo^8^, M. R. de la Torre^13^, G. Arana², K. Castro², J. M. Amigo², I. Martínez-Arkarazo², J. Aramendia², N. Prieto², I. Población², L. Coloma², F. Alberquilla², J. Huidobro², G. Gorla², F. Rull^7^, A. Sanz^7^, G. E. López-Reyes^7^, J. A. Manrique^7^, M. Veneranda^7^, I. Reyes^7^, S. Julve^7^, J. M. Vadillo^1,3^, J. Moros^1,3^, P. Lucena^1,3^, L. M. Cabalín^1,3^, T. Delgado^1^, M. Boskova^1^, L. García‑Gómez^1^, F. J. Fortes^1,3^, M. Cáceres^6^, J. Sánchez^6^, J. M. Cebriá^6^, Á. Lobato^6^, F. Izquierdo^6^, A. Inchausti^6^, D. Abbasi^6^, I. Serrano^6^, V. García-Baonza^6^, M. Herraiz-Sarachaga^9^, A. Delgado^14^, A. G. Moral^11^, A. Álvarez^11^, L. M. González^11^, M. Rodríguez^11^, G. Ramos^11^, M. Sanz^11^, D. Escribano^11^, J. F. Cabrero^11^, A. González-Fairén^8^, V. Parro^8^, F. Mansilla^8^, J. Pla-García^8^, L. Sánchez-García^8^, M. F. Sampedro^8^, M. R. López-Ramírez^15^, O. Bassy^13^, M. V. Ortega^13^, E. Cueto-Díaz^13^, S. Gálvez-Martínez^13^, M. P. Zorzano-Mier^13^, C. Pérez-Fernández^13^, P. Muñoz-Marzagón^12^, N. Bilbao^12^, D. Cortés^12^, N. González^12^, E. Izagirre^12^, M. Mayoral^7^, S. Jiménez-Blázquez^7^.

^1^UMALASERLAB, Departamento de Química Analítica, Universidad de Málaga, Málaga, Spain

^2^Department of Analytical Chemistry, University of the Basque Country (UPV/EHU), Leioa, Spain

^3^Instituto Universitario de Materiales y Nanotecnología, IMANA, Universidad de Málaga, Málaga, Spain

^4^ Central European Institute of Technology, Brno University of Technology, Brno, Czech Republic

^5^Faculty of Mechanical Engineering, Brno University of Technology, Brno, Czech Republic

^6^Department of Physical Chemistry, Universidad Complutense de Madrid (UCM), Madrid, Spain

^7^ERICA Research Group, University of Valladolid (UVa), Valladolid, Spain

^8^Centro de Astrobiología (CSIC-INTA), Madrid, Spain

^9^Instituto de Geociencias (IGEO, CSIC-UCM), Madrid, Spain

^10^Grupo de Hidrogeología, Departamento de Ecología y Geología, Facultad de Ciencias, Universidad de Málaga, Málaga, Spain

^11^Instituto Nacional de Tecnica Aeroespacial (INTA), Madrid, Spain

^12^Basque Centre for Climate Change (BC3), Leioa, Spain

^13^Departamento de Observación de la Tierra, Instituto Nacional de Técnica Aeroespacial (INTA)

^14^Stable Isotope Biogeochemistry Laboratory, IACT-CSIC, Granada, Spain

^15^Department of Physical Chemistry, Faculty of Science, University of Málaga, Malaga, Spain

**References**

[1] Shi, C., Wei, B., Wei, S., Wang, W., Liu, H., & Liu, J. AA quantitative discriminant method of elbow point for the optimal number of clusters in clustering algorithm. *EURASIP J. Wirel. Commun. Netw.* **2021**, 31 (2021).

[2] Onumanyi, A.J., Molokomme, D.N., Isaac, S.J.& Abu-Mahfouz, A.M. AutoElbow: an automatic elbow detection method for estimating the number of clusters in a dataset. *Appl. Sci.* **12**, 7515 (2022).

[3] Shutaywi, M. & Kachouie, N.N. Silhouette Analysis for Performance Evaluation in Machine Learning with Applications to Clustering. *Entropy* **23**, 759 (2021).
